# Structural and health system determinants of health outcomes in systemic lupus erythematosus: Understanding the mechanisms underlying health disparities

**DOI:** 10.3389/fpubh.2022.980731

**Published:** 2022-09-30

**Authors:** Jerik Leung, Lily McMorrow, Rhonda BeLue, Elizabeth A. Baker

**Affiliations:** ^1^Department of Behavioral, Social, and Health Education Sciences, Rollins School of Public Health, Emory University, Atlanta, GA, United States; ^2^Division of Rheumatology, Department of Medicine, Washington University School of Medicine, Saint Louis, MO, United States; ^3^Department of Public Health, University of Texas at San Antonio, San Antonio, TX, United States; ^4^Department of Behavioral Science and Health Education, College for Public Health and Social Justice, Saint Louis University, Saint Louis, MO, United States

**Keywords:** health disparities, chronic disease, systemic lupus erythematosus (SLE), structural determinants of health, health system, patient-provider interaction, subordination, structural competency

## Abstract

Chronic diseases are increasingly responsible for the burden of health outcomes across the world. However, there is also increasing recognition that patterns of chronic disease outcomes (e.g., mortality, quality of life, etc.) have inequities across race, gender, and socioeconomic groups that cannot be solely attributed to these determinants. There is a need for an organizing framework which centers fundamental causes of health disparities that may better guide future work in centering these mechanisms and moving beyond acknowledgment of health disparities. In this paper, we synthesize several concepts from health disparities literature into a conceptual framework for understanding the interplay of patients' lived experiences, the health care system and structural determinants. Our framework suggests that (1) structural factors influence the health care system, the patient, the health care provider, and the provider-patient relationship through process of subordination and (2) that structurally competent actions are critical to reducing health inequities. The addition of subordination to theoretical frameworks involving health equity and social determinants of health, along with engagement with concepts of structural competency suggest several systems level changes to improve health outcomes.

## Introduction

Chronic diseases are increasingly responsible for the burden of health outcomes across the world (1). Treatment of patients with chronic diseases primarily focuses on factors proximal to the individual (i.e., clinical markers), the provider-patient relationship, and the health care system. However, there is also increasing recognition that patterns of chronic disease outcomes (e.g., mortality, quality of life, etc.) have inequities across race, gender, and socioeconomic groups that cannot be solely attributed to these determinants (2). In this paper, we synthesize several concepts from health disparities literature into a conceptual framework for understanding the interplay of patients' lived experiences, the health care system and structural determinants in the context of the United States. Our framework suggests that (1) structural factors influence the health care system, the patient, the health care provider, and the provider-patient relationship through process of subordination and (2) that structurally competent actions are critical to reducing health inequities. We use our work with patients with systemic lupus erythematosus (SLE) and health care providers as an illustrative example. Without understanding the fundamental causes of disparities in SLE outcomes, we will continue to focus on individual level interventions (new therapeutics, behavioral interventions) and as a result may not meaningfully address existing disparities and could even exacerbate them (3). It is critical to understand these complex interactions to understand both where and how we can maximize improvements in patient quality of life and clinical outcomes.

## Health disparities in SLE

Despite low population prevalence (72.8 per 100,000) (4), there is a significant concern with health disparities both in the prevalence and outcomes among those with SLE. Specifically, 90% of people living with SLE are women, and Black women exhibit SLE rates nearly 4 times greater than other racial groups in the United States (5). In addition to these disparities in who has SLE, there is further evidence of disparities in both clinical and quality of life outcomes. For instance, a primary clinical outcome of interest is disease activity, defined as reversible evidence of immune activity. Previous studies have suggested that Black and Hispanic individuals exhibit higher rates of disease activity (5). Previous studies have also suggested disparities in downstream outcomes such as birth outcomes (worse for non-white women with SLE relative to general population) (6), cardiovascular events (younger age of hospital admission of Black women with SLE relative to both white women with SLE and general population) (7), and mortality (greater among Black individuals relative to white individuals) (8). Moreover, there is evidence of QOL—which captures individual perceptions of disease impact on everyday life across physical, and mental domains—disparities by race (9).

The mechanisms underlying these disparities in SLE are poorly understood but purely genetic or biological explanations are unlikely (10). Given the demographic differences in who has SLE, many groups in this field are focusing on social determinants of health (11–14). For instance, medication adherence is an area of concern among people living with SLE with some work suggesting Black individuals with SLE have lower adherence relative to white individuals (15). In this paper, we argue that in order for this type of observation, such as disparities in medication adherence, to contribute to concrete action in reducing health disparities, the reasons driving the observation must be contextualized into larger structural determinants (e.g., the context of patient-provider interactions, the interactions of marginalized people with the health care system, and socio-historical context).

## Structural determinants

The impact of structural determinants of health—categories of factors which create stratification in society—in shaping health outcomes is widely acknowledged (16). There is an abundance of evidence that supports the presence of gradients of health along factors such as discrimination and poverty (17), meaning that health progressively improves with level of societal advantage.

A potential process by which stratification and consequent gradient of health occurs is subordination (18). Subordination is, in our use of the term, “a set of practices, traditions, norms, definitions, cultural stories, and explanations” (18) that are used to justify the creation of systems and structures which function to “systematically hold down one social group to the benefit of another social group.” Subordination describes the process by which some groups of people (e.g., White, high-income) dominate others (e.g., Black, low-income) through differential access to power due to belonging to a certain social group. Subordination leads to the characteristics, cultures, and norms of those groups in power being reflected in institutions, systems, and structures in society, including health care systems, creating discordance for those not in power. This process may help explain the mechanisms through which socially constructed categories, such as race, can have real consequences on people's lives (i.e., poor health outcomes).

**Figure 1:**
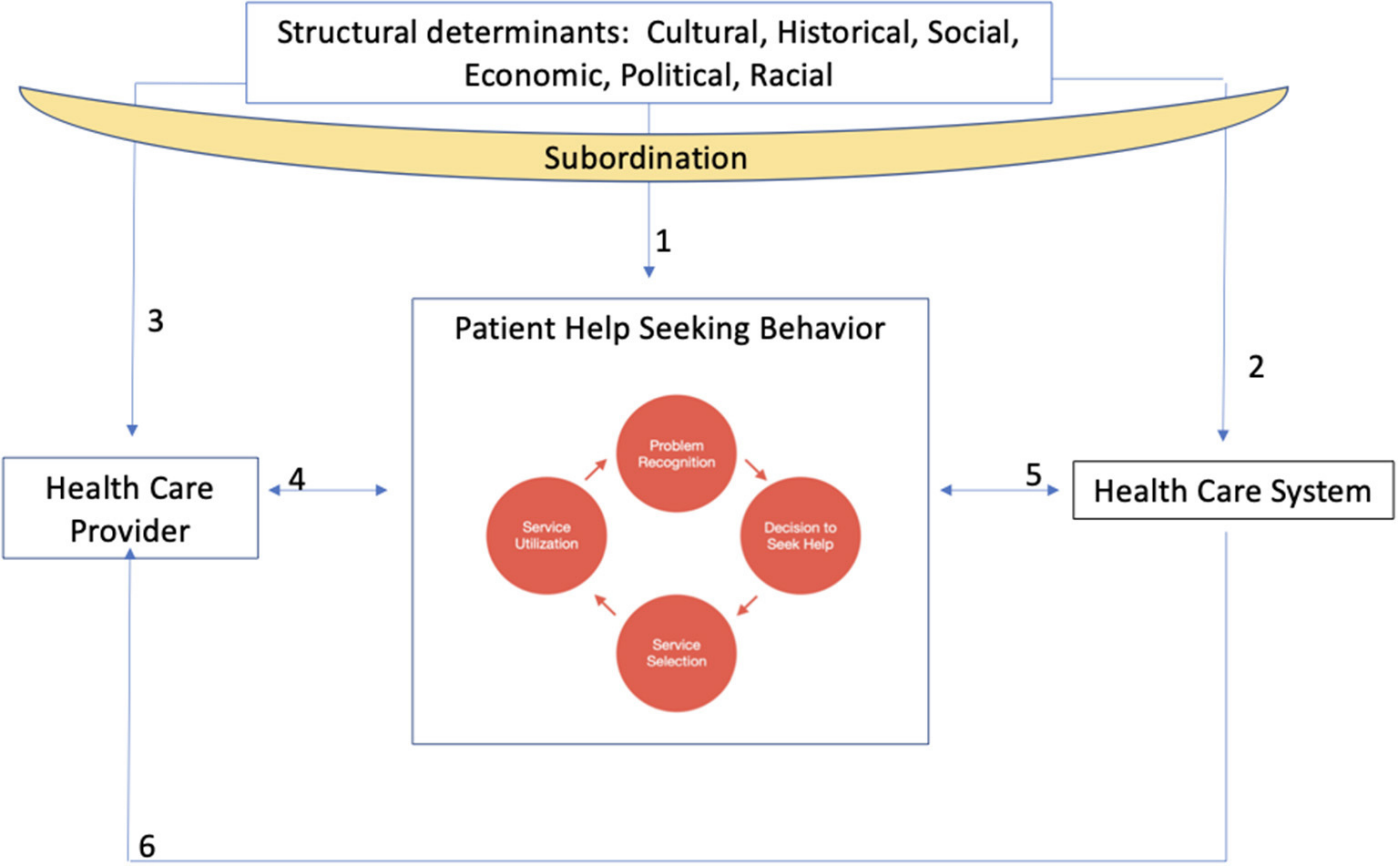
Conceptual framework depicting relationships between structural determinants, subordination, and health care providers, patients and the health care system.

What is critical for the current discussion is that these dominant group cultural norms are reflected and regularly reinforced in our institutions, systems, policies and structures (19). As institutional practices reflect the dominant cultural norms, this process affects which populations are best and least served by these institutions and shape the individual actors within those systems (Figure 1).

## Patient help-seeking behavior, engagement, and lupus

As illustrated in Figure 1, individuals cycle through help-seeking behavior over the disease course. Help-seeking behavior includes problem recognition, deciding to seek help, determining who can help, and using services (20). For patients, the recognition of a new symptom may prompt a decision to see a doctor who may provide referrals or different treatment options. Patients' previous interactions with their provider and the health care system influence these help-seeking behaviors (Figure 1, arrows 4 and 5) and patient engagement, which is the degree to which patients are actively involved with their care (21). Patients are, for example, influenced by structural factors and the impact of these on patients' experiences of discrimination/bias, community context, language, and social support systems across their life course may influence level of patient engagement and subsequent likelihood of entering help-seeking behavior cycle (Figure 1, arrow 1) (22). In addition, historical exploitation of marginalized populations at a societal level influences these behaviors because of direct effects on patient trust of providers and the health care system (Figure 1, arrows 2 and 3) (23). Moreover, the way that patients are perceived by the health care system and their health care provider(s) impacts patient help-seeking behavior and overall level of engagement with care (Figure 1, arrows 4 and 5) (24).

Patients with SLE demonstrate numerous challenges in beginning help-seeking behaviors and engagement with care (25), chief of which is the experience of uncertainty. This uncertainty can be precipitated by aspects of the disease such as flaring, the prolonged delay between symptoms and diagnosis (26) and variability of medication regimens (27). The variability of medications, side effects, and varying effectiveness can lead to patients doubting medication effectiveness and intentional non-adherence (28). Healthcare providers may react to these patient behaviors through reprimand or by minimizing patient concerns (29). This type of dynamic erodes trust between healthcare providers and patients (30), decreasing the likelihood that patients will actively participate in their care and associated help-seeking behaviors. These types of interactions may be further exacerbated by structural factors and the process of subordination stemming from experiences in health care resource utilization (e.g., patronizing interactions) and the ways these resources are organized (e.g., hours of operation).

## Health care providers

Health care providers are also shaped by their personal background, experiences of structural factors (Figure 1, arrow 3) and the health system (Figure 1, arrow 6). Health care providers are socialized as they progress through their medical education. In this process, they learn what is necessary to succeed in the health system, which in the United States often entails a transition from embodying humanist ideals of medicine toward technical expertise, and from valuing alternative perspectives of health and disease to focusing on Western notions of disease etiology and treatment (Figure 1, arrow 6) (31).

This socialization, coupled with a lack of diversification of the physician workforce (32), can manifest in communication difficulties between individuals of different identities and experiences. Cultural humility, defined as a lifelong learning process characterized by openness, self-awareness, self-reflection, and self-critique, is a useful concept to frame this issue (33). There is an abundance of literature citing lack of cultural humility among providers, ongoing deleterious effects of racism on black, indigenous, and other people of color (BIPOC) patients, and implicit bias favoring white race persons in medical school admissions (19, 34). As previous literature has examined, even for health care providers of color and other non-dominant groups, this can lead to negative outcomes for patients in that people of marginalized identities are viewed less favorably relative to those from dominant groups (35). Even while training has expanded in the medical field to address these issues, efforts often focus on cultural competency, which has been critiqued as perpetuating stereotypes and improperly communicating that culture can be “mastered” (33).

The experience of health care providers who specialize in SLE care demonstrates the socialization process and subsequent influences on patient care. For instance, health care providers desire to make evidence based clinical decisions but that available evidence is not always of high quality (36). This impacts patients through the difficulty in communicating uncertainty associated with SLE care and may decrease patient trust in the health care system (37). The impact of broader structural determinants and the process of subordination on this relationship is significant for patients with SLE. African Americans with SLE are more likely to experience hurried communication with providers, language that is difficult to understand, or a lack of respect, factors which may be associated with poorer medication adherence compared to their White counterparts (38). This example illustrates the process of subordination such that individuals in positions of power (often the health care provider) dictate the norms and time allotted for communication with patients, which in turn influences patient outcomes.

## Health care system

Patient help-seeking behaviors are influenced by health care system structures and processes (Figure 1, arrow 5) (39). Structure of care is defined as factors that prevent or facilitate provision of care and encompass the setting in which health care is provided as well as financial and organizational elements of the system (40). For example, recent work has suggested that highest quality of SLE care may come from specialized SLE clinics, which are often attached to academic medical centers (41). Given the disproportionate location of these institutions in urban centers, this highest quality care for someone living with SLE may not be accessible to those who are socioeconomically disadvantaged or do not have transportation to these medical centers (Figure 1, arrow 2) (42). Recent work also suggests that integrated care models may be particularly helpful in increasing the quality of chronic disease management (43). This variation in structure, and benefits of particular structures over others, suggests differential patient access to services.

Processes of care involve actions performed in giving and receiving care, including diagnoses and treatment. The health care system pressure to see high volumes of patients along with increased time spent on managing electronic medical records (Figure 1, arrow 6) reduces the time available per patient (44). These processes of care are salient to patients with SLE given the complex nature of the disease and myriad new symptoms or flares in existing symptoms that must be discussed at a given visit. These health system processes, which decrease the available time for patients and providers to interact, manifests as an increased strain on provider-patient relationships (Figure 1, arrow 4).

In our conception, the health care systems, structures, and processes operate both directly on the patient (Figure 1, arrow 5) and through the provider-patient interaction (Figure 1, arrow 4). Broader structural determinants including governmental systems, practices, laws, and policies also have direct impacts on the health care system, structures, and processes and in these ways also impact providers, patients, and the provider-patient relationship (Figure 1, arrows 2 and 3).

These structures and processes operate in a larger context of a shift in United States health care toward the chronic care model (CCM) (45). The CCM is framework designed to assist health care organizations to reorient services from a prioritization of acute care of symptoms toward health promoting and preventive care (45). While the implementation of CCM in SLE management settings would stand to benefit patients (12), there are several challenges such as the often delayed diagnosis, still developing self-management and other non-pharmacologic interventions (46). and need for tailored information and techniques which align with symptom fluctuations (47).

The health system is itself shaped by structural determinants through subordination. For instance, the mode of health care delivery and larger conception of health care as a commodity contributes to disparities in who can obtain these goods and services. Moreover, the power and decision making of health care systems are often reflective of dominant identities in society (White, male, heterosexual, cisgender, able), which translate into a health care delivery system that most optimally benefits those dominant groups (48).

## Health care provider-patient interactions

The interaction of health care providers and patients is the culmination of these factors and takes place within the web of the healthcare system (Figure 1, arrow 4). Health care providers have their own background characteristics that may influence their motivations in medicine but are also socialized into a biomedical system which values technical aptitude, often over patient perspectives. A patient's health care history is shaped by previous experiences with health care providers and structural barriers to care which may manifest as negative attitudes toward the health care system which may in turn become the lens through which the patient views the provider and influences their decision-making. These interactions with the health system and providers contribute to a patient's trust in the health care system (49).

For patients with SLE these interactions occur across many years and with many providers (26). Patients often struggle to communicate their symptoms to their provider and can face resistance or experience a lack of validation that what they are experiencing is “real” and related to their SLE (30). Structural determinants through subordination can also shape this experience due to the power imbalance in the interaction and the societal prioritization of physician-expert knowledge over the patient perspective.

## Discussion

Individual patient help-seeking behaviors are shaped by the health care system structures and processes, interactions which are also influenced by structural factors through subordination. Moreover, current behavior is influenced by patients' personal, social and historical experiences of marginalization and past experiences with the health care system and providers.

While our model is consistent with previous discussions of social determinants and health (16, 50). the novelty of this work is integration of a linking concept (subordination) between individual, interpersonal factors, and structural factors (e.g., patients and their interactions with their health care providers and the health system). There is momentum in engaging with these determinants, but conceptual frameworks often lack pathways which capture interaction among levels. The concept of subordination offers a foundation on which to construct pathways which link outer structural determinants to individual health outcomes.

This commentary speaks to the importance of not only stating that there are structural factors influencing health disparities but understanding the way that these factors influence health outcomes and intervening to affect change in education and clinical practice. Structural competency training is an action suggested by our framework. Structural competency has been defined as the “ability to discern how a host of issues defined clinically as symptoms, attitudes, or diseases also represent the downstream implications of a number of upstream decisions…” (51) without the structural viewpoint there may be a tendency to focus interventions on one specific link in the system (i.e., patient-provider interactions) without acknowledging the interrelatedness of all system concepts (i.e., structural determinants of health and subordination). By increasing understanding of the impact of structural factors, we can envision the changes needed to address fundamental causes of disparities and to ensure that systems and structures are created in ways that work equitably for everyone. An example of current changes which could better address health disparities through structurally competent framing are integrated clinical care models, which may better serve patients by engaging multiple types of health care providers and addressing symptom management along with access to resources needed to engage in behavioral change (43). Another example is diversifying the healthcare workforce throughout the medical hierarchy, a process which is particularly needed in rheumatology given the racial homogeneity in the profession (52) and which can translate into better outcomes for patients from marginalized groups (19).

The proposed framework also has implications regarding guiding future research. A structurally competent approach enables us to better contextualize the purpose of research from bench to bedside, guiding the types of questions asked and ensuring that the questions incorporate the impact of structural factors. A structurally competent approach may also help to unveil the factors contributing to persistently low participation of racial minority groups in randomized clinical trials for not only SLE research (53).

The inclusion of these elements in our conceptual framework does not imply that these are the only factors or relationships that impact health outcomes for patients. We have presented this framework, developed with SLE patients in mind and grounded in previous work exploring unmet needs and the lived experience of SLE patients (28, 30), to provide an example of how mapping out these elements for a particular population can help identify leverage points for action. In addition, this model was conceived from a perspective of the United States and we considered variations in major demographic categories in that lens. Future work would need to consider how this model may differ across different types of cultural contexts and health care systems but we encourage people who work in other contexts to follow this process of explicitly stating linkages between structural determinants and their health outcomes of interest. Lastly, this conceptual framework has focused on structural determinants which shape patient behaviors but is important to recognize that individuals also have agency in their decision-making and it is certainly influenced by things outside of what we discussed here (e.g., family networks, media consumption).

These systems changes require time and commitment from organizational leadership. There is evidence that institutions are committing to systemic changes which aim to be actively inclusive toward more people by adopting a justice-oriented approach to health care delivery (54). It is important that these types of efforts are coupled with an evaluation approach that can can measure the extent to which these types of systems changes lead to meaningful outcomes for patients (55). Developing both areas in future work, guided by a framework which centers structural determinants as a fundamental cause, will be key to making meaningful progress in addressing health disparities.

## Data availability statement

The original contributions presented in the study are included in the article/supplementary material, further inquiries can be directed to the corresponding author.

## Author contributions

JL authored the first draft with LM, RB, and EB all contributing to reviewing and editing of the original and subsequent drafts. RB and EB provided senior supervision. All authors contributed to conceptualization of the manuscript.

## Funding

JL was supported by the Saint Louis University Office of Graduate Education. EB was supported by a grant from the Robert Wood Johnson Foundation Interdisciplinary Research Leaders Fellows program.

## Conflict of interest

The authors declare that the research was conducted in the absence of any commercial or financial relationships that could be construed as a potential conflict of interest.

## Publisher's note

All claims expressed in this article are solely those of the authors and do not necessarily represent those of their affiliated organizations, or those of the publisher, the editors and the reviewers. Any product that may be evaluated in this article, or claim that may be made by its manufacturer, is not guaranteed or endorsed by the publisher.
